# Hypoglycemia measured by flash glucose monitoring system predicts liver-related events in chronic liver disease patients

**DOI:** 10.1038/s41598-023-40910-2

**Published:** 2023-08-23

**Authors:** Ryu Sasaki, Naota Taura, Yasuhiko Nakao, Masanori Fukushima, Masafumi Haraguchi, Satoshi Miuma, Hisamitsu Miyaaki, Kazuhiko Nakao

**Affiliations:** Department of Gastroenterology and Hepatology, Nagasaki University Graduate School of Biomedical Sciences, 1-7-1 Sakamoto, Nagasaki City, Nagasaki, 852-8501 Japan

**Keywords:** Hepatology, Liver, Liver diseases, Diabetes

## Abstract

Impaired glucose tolerance, glucose fluctuations, and hypoglycemia have been observed in patients with chronic liver disease (CLD). The flash glucose monitoring (FGM) system, which recognises continuous and dynamic glucose changes in real time, is used in daily clinical practice. This study aimed to examine the association between glucose fluctuations and hypoglycemia, as measured by the FGM system, and liver-related events. Seventy-two patients with CLD and type 2 DM who had their blood glucose measured using Freestyle Libre Pro between April 2017 and July 2018 at our institution were enrolled in this retrospective study. We assessed the results of the FGM system measurements and liver-related events, as defined by gastrointestinal bleeding, infection, ascites, encephalopathy, and liver-related death. The standard deviation (SD) of mean glucose as measured by the FGM system was 41.55 mg/dl, and hypoglycemia was observed in 48.6% (35/72) of the patients. Liver-related event-free survival was not significant when stratified based on SD; however, the event-free survival was significantly lower when stratified by hypoglycemia (*p* = 0.007). In a multivariate analysis using the Cox proportional hazards model, Child–Pugh class B [Hazards ratio (HR) 2.347 (95% confidence interval (CI): 1.042–5.283), *p* = 0.039] and hypoglycemia [HR 2.279 (95% CI: 1.064–4.881), *p* = 0.034] were identified as factors contributing to event-free survival. Hypoglycemia, as determined by the FGM system, was identified as a significant factor that was closely associated with liver-related events. In addition to measuring glucose levels, the FGM system is useful in predicting the occurrence of liver-related events.

## Introduction

Impaired glucose tolerance is frequently observed in patients with chronic liver disease (CLD)^1^ and diabetes is an independent risk factor for CLD^2,3^. Patients with diabetes mellitus (DM) and CLD, especially those with liver cirrhosis (LC), are likely to experience both fasting hypoglycemia and postprandial hyperglycemia^4^. Glycemic dynamics in patients with CLD are characterized by marked glycemic fluctuations^5^. However, the relationship between blood glucose dynamics and the prognosis of patients with CLD is not well known.

Unlike the traditional self-monitoring of blood glucose, the continuous glucose monitoring (CGM) system measures interstitial glucose levels to recognize continuous and dynamic glucose changes in real time over 24 h. We reported the association between glycemic fluctuations using the CGM system (iPro2, Medtronic, Northridge, CA, United States) and sleep disorders in a previous study^6^.

In addition to the CGM system, the flash glucose monitoring (FGM) system has recently become commercially available and is being used in daily clinical practice. The FGM system (FreeStyle Libre Pro, Abbott Japan LLC, Tokyo, Japan) is a factory-calibrated, commercially available sensor system. This eliminates the necessity of capillary glucose testing for sensor calibration, thus improving convenience. Unlike the conventional CGM system, the FGM system is capable of long-term measurements and provides more detailed information on blood glucose dynamics.

It is predicted that the FGM system will be able to detect glucose fluctuations and hypoglycemia which are not obvious in patients with CLD. The purpose of this study was to verify whether the FGM system can detect glucose fluctuations and hypoglycemia and to determine whether it has any effect on liver-related events.

## Materials and methods

### Patients

Seventy-two patients with CLD and type 2 DM who had their blood glucose measured using FreeStyle Libre Pro between April 2017 and July 2018 at our institution were enrolled in this retrospective study.

The following cases were excluded from the analysis: sensor data of < 24 h (n = 3), sensor loss due to unplanned removal (n = 1), and sensor malfunction with unrecorded data (n = 1).

### Diagnosis of DM and liver cirrhosis

Type 2 DM was diagnosed based on a fasting plasma glucose (FPG) level > 126 mg/dL or plasma glucose > 200 mg/dL at 120 min after oral glucose loading or HbA1c > 6.5%. Patients were clinically divided into chronic hepatitis and LC groups. LC was defined based on Fibroscan > 12.5 kPa.

### Measurement of FGM

Subcutaneous interstitial glucose levels were measured using the FGM system for up to 14 days. The sensor was inserted into the subcutaneous tissue of the upper arm and continuous sensor values were recorded every 15 min until it was removed. The measurement results automatically stored in the sensor were wirelessly transferred to the reader and analysed using the FreeStyle Libre Pro Software.

### Definition of glycemic parameters by FGM

The standard deviation (SD) of mean glucose, mean amplitude of glycemic excursions (MAGE), time in tight range (70–140 mg/dL), time below range (< 70 mg/dl), time above range (> 180 mg/dl), mean sensor glucose, and coefficient of variation (CV; calculated as 100 × SD divided by mean glucose) were calculated from the records in accordance with an international consensus statement^7^. Hypoglycemia was defined as less than 70 mg/dl for at least 15 consecutive minutes that occurred more than once. MAGE is a classical index used to quantify large fluctuations in blood glucose levels. It represents the arithmetic mean of the difference between the highest and lowest continuous blood glucose levels over 24 h, when the difference is more than 1 SD from the mean blood glucose level^8,9^.

### Follow-up and diagnosis of liver-related events

We identified several clinical endpoints including gastrointestinal bleeding, infection, ascites, encephalopathy, and liver-related death. Clinical progression was defined as the onset of these events. Patients who underwent liver transplantation were censored at the time of transplantation. Follow-up was continued every 1–3 months and on the date of hospitalization. The observation period lasted until April 2021.

### Ethical considerations

Informed consent for the use of the medical records was obtained from each patient. The study protocol was approved by the Ethical Committee of our institution (Approval Number: 17082127) and conformed to the 1975 Declaration of Helsinki and the Japanese Ethical Guidelines for Clinical Research (Ministry of Health, Labour, and Welfare of Japan, Ethical Guidelines for Clinical Research, 2008).

### Statistical analysis

Continuous variables (age, body mass index, duration of DM, platelet count, alanine transferase, FPG, immunoreactive insulin, HbA1c, glycoalbumin, SD of mean glucose, CV, and MAGE) were dichotomized with respect to the median or clinically meaningful values in a multivariate analysis. Spearman’s rank correlation coefficient was used for statistical analysis, and the Mann–Whitney U test was used for comparison between the two groups. The Kaplan–Meier method and log-rank test were used to estimate the event-free survival rate. Cox proportional hazards regression analysis was performed to evaluate the risk factors for event-free survival. Statistical significance was set at *p* < 0.05. Data analysis was performed using SPSS ver. 22.0 (SPSS, Chicago, IL, USA).

## Results

### Patient characteristics

The baseline characteristics of the 72 patients included in this study are summarized in Table 1. The median duration of diabetes was 9.0 years. All patients received glucose-lowering therapy and 25.0% used insulin. Overall, 88% of the patients had cirrhosis and 48.6% had a history of liver cancer. The median observation period was 35.0 months.

**Table 1:** Characteristics of the patients.

Variables	n = 72
Age (years)	67.5 (61.5–76.0)
Sex (male/female)	49/23
BMI (kg/m^2^)	24.15 (21.60–28.45)
Diabetic duration (year)	9.0 (3.0–14.5)
History of hepatocellular carcinoma (yes/no)	35/37
Insulin therapy (Basal plus bolus/bolus alone/basal alone/no)	10/4/4/54
Glucose-lowering therapy (Metformin/Sulfonylureas/DPP-4 inhibitors/GLP-1 agonists/others)	5/6/37/4/23*
BCAA (yes/no)	23/49
Etiology (HBV/HCV/alcohol/NBNC)	7/2016/29
Performance status 0/1/2/3	50/9/7/6
Liver cirrhosis (yes/no)	37/35
Child–Pugh class (A/B)	48/24
Platelet count (× 10^4^/μl)	12.90 (8.85–18.85)
PT (%)	85.0 (72.5–96.0)
T.bil (mg/dl)	1.00 (0.70–1.45)
Albumin (g/dl)	3.55 (3.05–3.90)
ALT (IU/ml)	24.0 (16.0–50.5)
FPG (mg/dl)	122.5 (104.0–154.5)
IRI (μU/ml)	11.35 (5.75–20.00)
HbA1c (%)	6.60 (5.90–7.45)
Glycoalbumin (%)	20.30 (17.25–22.30)

Data are given as the medians with interquartile ranges or numbers.

*Includes duplicate cases.

*BMI* body mass index, *DPP-4* dipeptidyl peptidase-4, *GLP-1* glucagon-like peptide-1, *PT* prothrombin, *T.bil* total bilirubin, *ALT* alanine aminotransferase, *FPG* fasting plasma glucose, *IRI* immunoreactive insulin, *HbA1c* hemoglobin A1c.

### Glucose parameters and FGM parameters

**Figure 1:**
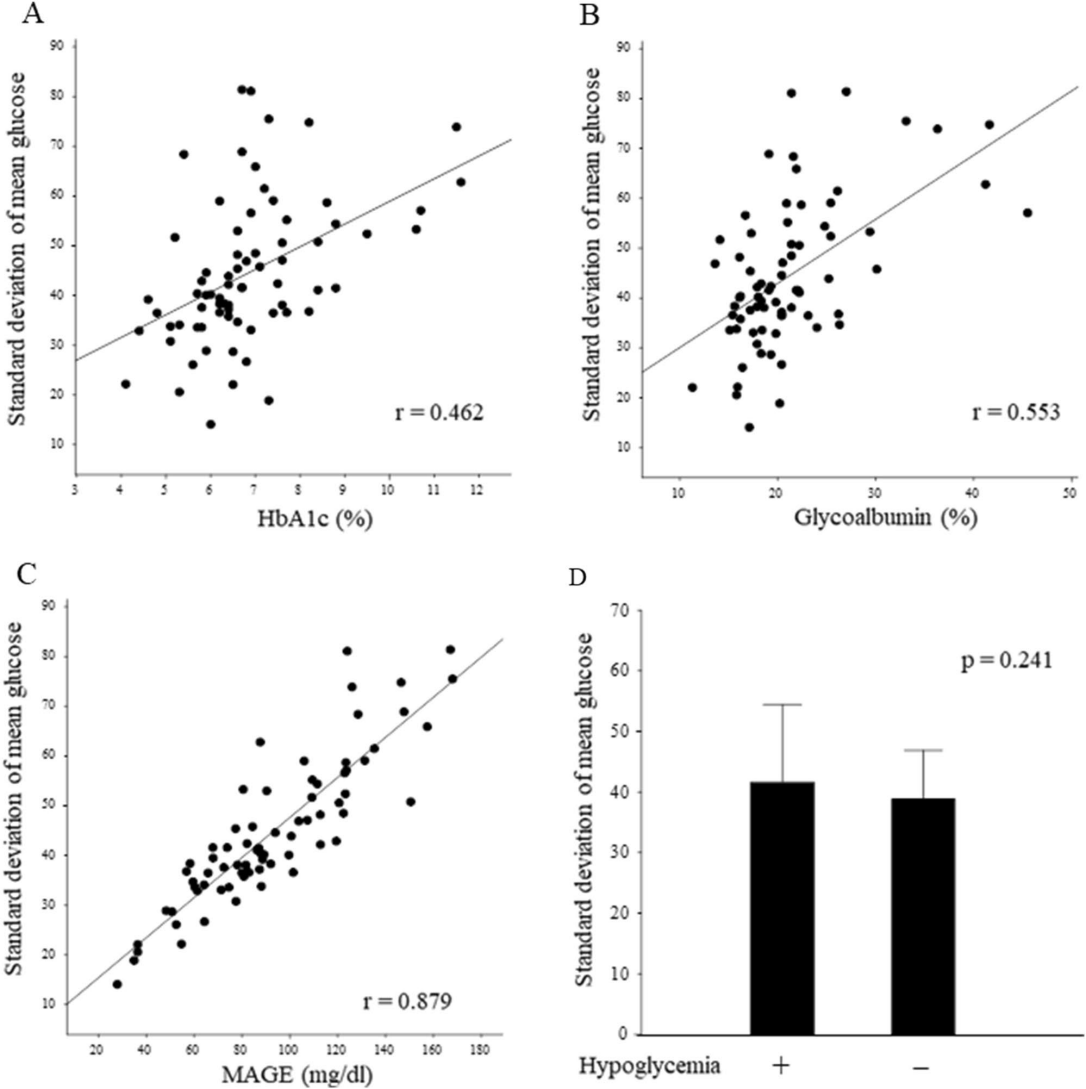
Association of the standard deviation of mean glucose with classical DM control index and hypoglycemia. The standard deviation of mean glucose correlated with HbA1c (**A**), Glycoalbumin (**B**), and MAGE (**C**). (**D**) Standard deviation of mean glucose did not differ between patients with and without hypoglycemia.

The results of glucose metabolism using peripheral blood measurements and the FGM system are shown in Table 2. The SD of mean glucose level as measured by the FGM system was 41.55 mg/dl and median CV was 29.6%, and hypoglycemia was observed in 48.6% (35/72) of the patients. The median percentage of time below range was 1.0% overall and 4.5% for the hypoglycemia group only. In addition, 55.0% of the measured hypoglycemic events were detected in the nocturnal period (0000–0559 h). There was a correlation between classical glycemic variability parameters and SD values (Fig. 1). The correlation between HbA1c and glycoalbumin was weak (Fig. 1A and B), while MAGE showed a strong correlation (Fig. 1C). However, no significant difference was observed between hypoglycemia and SD values (*p* = 0.241, Fig. 1D).

**Table 2:** Laboratory characteristics associated with flash glucose monitoring.

Variables	n = 72
Flash glucose monitoring	
Time in tight range (%)	74.5 (59.0–86.5)
Time below range (%)	1.0 (0.0–2.0)
Time above range (%)	20.5 (10.5–40.0)
Mean sensor glucose (mg/dl)	142.5 (119.0–173.0)
Coefficient of variation (%)	29.6 (24.7–34.7)
Hypoglycemia (yes/no)	35/37
Standard deviation of mean glucose (mg/dl)	41.55 (36.15–53.15)
MAGE (mg/dl)	87.75 (69.60–116.10)

Data are given as the medians with interquartile ranges or numbers.

*MAGE* mean amplitude of glycemic excursions.

### Incidence rates of liver-related events

**Figure 2:**
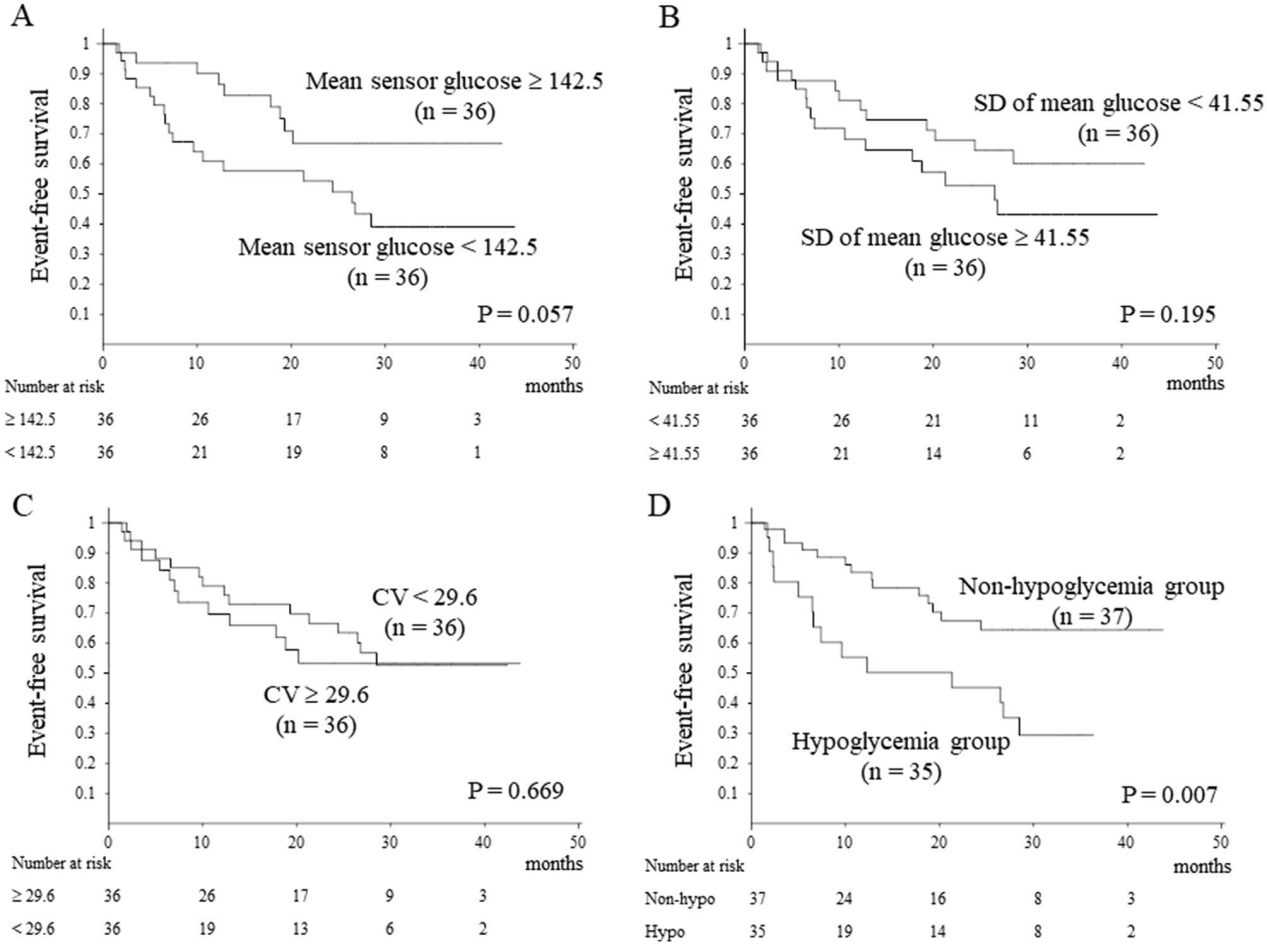
Liver-related event-free survival. No significant difference was seen (**A**, **B**, **C**). Liver-related event-free survival based on glucose levels showed significantly lower event-free survival in the hypoglycaemia group (*p* = 0.007, **D**).

During the observation period, five patients underwent liver transplantation. There were 28 liver-related events and the event-free survival rate at 1 year was 74.6%. Liver-related events included encephalopathy in nine cases, ascites in eight cases, gastrointestinal bleeding in seven cases, and severe infection in four cases. Liver-related event-free survival was compared between the two groups according to mean sensor glucose, SD of mean glucose, and CV values, but no significant difference was found (Fig. 2A, B, C). Contrarily, liver-related event-free survival according to glucose levels showed significantly lower event-free survival in the hypoglycemia group (*p* = 0.007, Fig. 2D).

### Multivariate analysis of factors contributing to liver-related event-free survival

Table 3 shows the multivariate analysis of factors contributing to event-free survival using the Cox proportional hazards model. In univariate analysis, branched-chain amino acids (BCAA), Child–Pugh class, HbA1c, and hypoglycemia were identified as factors contributing to event-free survival. In multivariate analysis, Child–Pugh class B (hazard’s ratio (HR) 2.347 [95% confidence interval (CI): 1.042–5.283], *p* = 0.039) and hypoglycemia (HR 2.279 [95% CI: 1.064–4.881), *p* = 0.034) were identified as factors contributing to event-free survival.

**Table 3:** Multivariate analysis of factors contributing to event-free survival using the Cox proportional hazards model.

Factor		Univariate analysis	*P* value	Multivariate analysis	*P* value
		HR (95%CI)		HR (95%CI)	
Age	> 67.5 years	1.142 (0.539–2.417)	0.729		
Sex	Male	1.616 (0.756–3.454)	0.215		
BMI	> 24.15 kg/m^2^	0.904 (0.430–1.901)	0.790		
Diabetic duration	> 9.0 years	0.758 (0.358–1.603)	0.468		
History of hepatocellular carcinoma	Yes	1.917 (0.864–4.251)	0.109		
Insulin therapy	Yes	1.151 (0.489–2.709)	0.747		
Glucose-lowering therapy	Yes	0.852 (0.345–2.106)	0.728		
BCAA	Yes	2.741 (1.300–5.781)	0.008	1.793 (0.816–3.939)	0.145
Etiology	NBNC	0.864 (0.399–1.872)	0.710		
Performance status	1/2/3	1.505 (0.661–3.426)	0.330		
Liver cirrohsis	Yes	1.983 (0.831–4.728)	0.122		
Child–Pugh class	B	2.672 (1.230–5.801)	0.012	2.347 (1.042–5.283)	0.039
Platelet count	< 12.9 × 10^4^/μl	1.589 (0.750–3.367)	0.226		
ALT	> 24 IU/ml	0.832 (0.396–1.751)	0.628		
FPG	> 122.5 mg/dl	0.640 (0.300–1.368)	0.249		
IRI	> 11.35 μU/ml	2.054 (0.959–4.398)	0.063		
HbA1c	> 6.6%	3.206 (1.468–7.002)	0.003	2.251 (0.972–5.212)	0.058
Glycoalbumin	> 20.3%	0.798 (0.379–1.678)	0.551		
Hypoglycemia	Yes	2.661 (1.267–5.589)	0.009	2.279 (1.064–4.881)	0.034
Mean sensor glucose	> 142.5 mg/dl	0.439 (0.198–1.072)	0.052		
SD of mean glucose	> 41.55 mg/dl	0.608 (0.286–1.294)	0.196		
Coefficient of variation	≥ 29.6%	0.849 (0.402–1.792)	0.668		
MAGE	> 87.75	0.739 (0.351–1.555)	0.424		

*BMI* body mass index, *ALT* alanine aminotransferase, *FPG* fasting plasma glucose, *IRI* immunoreactive insulin, *HbA1c* hemoglobin A1c, *SD* standard deviation, *MAGE* mean amplitude of glycemic excursions.

## Discussion

Hepatic glycogen storage is decreased in patients with CLD^10^. Postprandial glucose uptake from the blood by the liver is delayed, resulting in hyperglycemia. In addition, patients with CLD have a reduced storage capacity for hepatic glycogen, resulting in inadequate glucose release from the liver into the blood during fasting, and impaired gluconeogenesis, leading to hypoglycemia; therefore, the management of diabetes in patients with liver disease can be difficult^11^. The metabolic state of patients with CLD after an overnight fast is similar to that observed in healthy individuals after 2–3 days of starvation. For this reason, patients with CLD have large fluctuations in blood glucose levels, and nocturnal hypoglycemia often occurs.

In the analysis focusing on glucose fluctuation, there was a weak correlation with DM parameters such as HbA1c and GA, and a strong correlation with MAGE, a classical fluctuation parameter (Fig. 1). The SD of mean glucose level as measured by FGM can be considered a surrogate index for MAGE. The CV was also examined as a measure of fluctuation; however, SD had a stronger correlation with MAGE. In our previous study, the SD was 24.1 mg/dl^6^, and it was reported to be approximately 14 in healthy subjects^12^. In the present study, the SD value of blood glucose was 41.5 mg/dl, which was as high as expected and considered to be affected by CLD. There was no significant relationship between glucose fluctuations and liver-related events.

The CGM system has been reported to be useful in detecting hidden abnormalities in blood glucose fluctuations in patients with type 2 DM and CLD^13^. Abnormal blood glucose fluctuations have also been reported to be a risk factor for sleep disturbance and decreased quality of life in patients with LC^6^. However, glucose fluctuation does not predict liver-related events such as encephalopathy, infection, and liver failure, which are more severe in patients with CLD. Similar results indicating that hypoglycemia is more important than blood glucose fluctuations have been reported, although not in patients with liver disease^14^.

In this study, hypoglycemia, as determined by the FGM system, was identified as a significant factor closely associated with liver-related events. Hypoglycemia plays an important role in inflammation, thrombotic events, and endothelial dysfunction by inducing oxidative stress^15^. Previous reports have shown that hypoglycemia is an important prognostic factor for short-term mortality in patients with cirrhosis ^16^. In addition, hypoglycemia has been reported to be associated with nutritional deficiencies, infections, and poor glucogenesis^17,18^. The presence of DM itself is associated with infections, variceal hemorrhage, and encephalopathy^19,20^. Previous reports have consistently shown that hypoglycemia is an important factor in liver-related events. Therefore, it is reasonable to accept hypoglycemia as a risk factor for liver-related event-free survival in our study.

Hypoglycemia measured using the FGM system showed a higher frequency in the total patient population (48.6%, 35/72). Previous studies of continuous blood glucose measurement using iPro2 have shown that hypoglycemia is infrequent (16.3%)^6^. Compared to iPro2, FGM has a long measurement period of up to 14 days, making it easier to detect hypoglycemia. It has been reported that CGM systems using FreeStyle Libre Pro can detect hypoglycemia better than point-of-care capillary glucose testing^21^. In addition, glucose measurements using this CGM system have been reported to be slightly lower than blood glucose levels^21^. Furthermore, 55.0% of the measured hypoglycemic events were detected during the nocturnal period, indicating that FGM is superior in detecting latent hypoglycemia and has a higher hypoglycemic frequency than previously reported.

Glycated hemoglobin A1c (HbA1c) and glycoalbumin are the gold standard indicators of glycemic control in diabetes. However, HbA1c cannot adequately represent the glycemic control status in patients with CLD because of the short lifespan of erythrocytes caused by hypersplenism. Glycoalbumin is affected by impaired albumin metabolism; in patients with CLD, the half-life of serum albumin is prolonged owing to decreased albumin synthesis^22^. Therefore, it is difficult to accurately monitor the glycemic control status in patients with CLD. The FGM system has enabled the identification of hypoglycemia in patients with CLD at a high risk of liver-related events. It is an excellent system for detecting latent hypoglycemia during a routine examination in a population with apparently good glycemic control, including in those with low HbA1c and glycoalbumin levels.

Late evening snack (LES) with BCAA supplementation is considered to be effective in improving protein-energy nutrition^23^ and avoiding nocturnal hypoglycemia in patients with CLD. In this study, BCAA and LES with BCAA were administered to 31.4 and 4.2% of the patients, respectively. Since all patients had type 2 DM, the possibility that they avoided calorie intake was considered, and there is a potential opportunity for intervention in the future.

A limitation of this study is that it was a single-centre retrospective analysis and there were no treatment interventions based on the FGM system measurements. Whether an intervention for hypoglycemia with FGM leads to a reduction in liver-related events is unknown^24^ and is a subject for future research. Another limitation is that the FGM system measurements were performed under health insurance and patients with CLD without therapeutic intervention for DM were not monitored by the FGM system.

Despite these limitations, this is the first report to describe the relationship between hypoglycemia identified using the FGM system and liver disease-related events. These results suggest that the FGM system, in addition to measuring glucose levels, is useful for predicting the occurrence of liver-related events.

## Data Availability

The datasets used and/or analysed during the current study available from the corresponding author on reasonable request.

## Abbreviations

CLD: Chronic liver disease
DM: Diabetes mellitus
LC: Liver cirrhosis
SMBG: Self-monitoring of blood glucose
CGM: Continuous glucose monitoring
FGM: Flash glucose monitoring
SD: Standard deviation
FPG: Fasting plasma glucose
MAGE: Mean amplitude of glycemic excursions
BMI: Body mass index
ALT: Alanine aminotransferase
IRI: Immunoreactive insulin
HbA1c: Hemoglobin A1c
BCAA: Branched chain amino acid
LES: Late evening snack
